# Metabolic anchor reactions for robust biorefining

**DOI:** 10.1016/j.ymben.2017.02.010

**Published:** 2017-03

**Authors:** Paula Jouhten, Jaime Huerta-Cepas, Peer Bork, Kiran Raosaheb Patil

**Affiliations:** a European Molecular Biology Laboratory, Heidelberg, Germany; b Molecular Medicine Partnership Unit, University of Heidelberg and European Molecular Biology Laboratory, 69117 Heidelberg, Germany; c Max Delbrück Centre for Molecular Medicine, 13125 Berlin, Germany; d Department of Bioinformatics, Biocenter, University of Würzburg, 97074 Würzburg, Germany

**Keywords:** Cell factory, Growth-product coupling

## Abstract

Microbial cell factories based on renewable carbon sources are fundamental to a sustainable bio-economy. The economic feasibility of producer cells requires robust performance balancing growth and production. However, the inherent competition between these two objectives often leads to instability and reduces productivity. While algorithms exist to design metabolic network reduction strategies for aligning these objectives, the biochemical basis of the growth-product coupling has remained unresolved. Here, we reveal key reactions in the cellular biochemical repertoire as universal anchor reactions for aligning cell growth and production. A necessary condition for a reaction to be an anchor is that it splits a substrate into two or more molecules. By searching the currently known biochemical reaction space, we identify 62 C‐C cleaving anchor reactions, such as isocitrate lyase (EC 4.1.3.1) and L-tryptophan indole-lyase (EC 4.1.99.1), which are relevant for biorefining. The here identified anchor reactions mark network nodes for basing growth-coupled metabolic engineering and novel pathway designs.

## Highlights

• Biochemical basis of growth-product coupling pinpointed.
• Anchor reactions are necessary for growth-product coupling.
• 62 biotechnologically relevant anchors identified.

## 1 Introduction

Engineered microbial cells offer sustainable production platforms for a large number of industrially important molecules. The production pathways in such cell factories inevitably need to divert the up-taken carbon away from the cell growth. Owing to this competition, microbial production strains harbor an inherent risk for instability leading to the loss of production capability (Van Dien, 2013). An elegant solution to this problem would be to align, through modifications in the host metabolism, the engineering objective (i.e. production flux) with the biological objective (i.e. cell growth) (Burgard et al., 2003, Patil et al., 2005). The main idea in this growth-product coupling is to reduce the possibilities that a cell has for synthesizing building block metabolites, e.g. an amino acid, such that the remaining route(/s) release the desired target molecule as a by-product. Indeed, this strategy has successfully been demonstrated in two industrially important hosts – *Escherichia coli* and *Saccharomyces cerevisiae* (Lee et al., 2005, Ng et al., 2012, Otero et al., 2013, Tokuyama et al., 2014). While several algorithms exist to aid the identification of gene deletion strategies to create growth-product coupling (Burgard et al., 2003, Patil et al., 2005, Klamt and Mahadevan, 2015), the biochemical basis of the coupling is yet unresolved. Here, we establish anchor reactions as fundamental biochemical links enabling growth-product coupling.

## 2 Methods

**Fig. 1:**
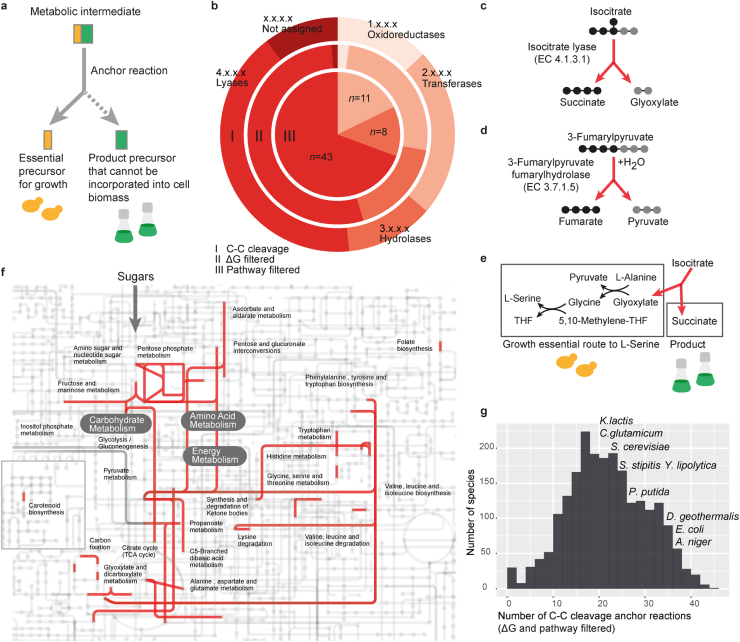
Metabolic anchor reactions for robust growth-product coupling. (**a**) Basic concept of an anchor reaction. Actual anchor reactions often include additional substrates and/or products. (**b**) EC classifications of the potential C-C cleaving anchor reactions in a universal biochemical reaction database KEGG (Kanehisa and Goto, 2000, Kanehisa et al., 2016): a total of 223 C-C cleaving anchor reactions (I, outer pie), out of which 97 were found to be thermodynamically feasible (II, middle pie), and a final set of 62 anchors after filtering of toxin and biomass component cleaving reactions (Supplementary information) (III, inner pie). (**c**) Isocitrate lyase is an example of a lyase C-C cleaving anchor reaction. Isocitrate lyase cleaves isocitrate (a six carbon compound) into succinate (four carbon compound) and glyoxylate (a two carbon compound). (**d**) 3-Fumarylpyruvate fumarylhydrolase is an example of a hydrolase C-C cleaving anchor reaction. It cleaves 3-fumarylpyruvate into fumarate and pyruvate. (**e**) Isocitrate lyase anchors an experimentally validated growth coupled production of succinate in *S. cerevisiae* (Otero et al., 2013). (**f**) The final set of 62 biotechnologically relevant C-C cleaving anchor reactions are highlighted in red on a global map of known metabolism using iPath v2 (Letunic et al., 2008, Yamada et al., 2011). (**g**) Prevalence of 62 biotechnologically relevant C-C cleaving anchor reactions in prokaryotes and fungi cataloged in the KEGG database (rel. 78 Apr 1st, 2016) (Kanehisa and Goto, 2000, Kanehisa et al., 2016). The strain representing *E. coli* indicated in the histogram is *E. coli* K-12 MG1655 and the strain representing *Pseudomonas putida* is *P. putida* KT2440. Other species specifically indicated are represented by the corresponding single strains available in KEGG database (rel. 78 Apr 1st, 2016).

We define an anchor reaction as a reaction that splits a substrate molecule to form two or more products (or parts of them) (Fig. 1a). Consider a metabolic network that does not include any anchor reactions. Any steady-state flux determination problem in such a network simplifies to a network flow problem in a directed graph (with metabolites as nodes and reactions as edges). Since any network flow problem in this graph can be reduced to one with exactly one source metabolite and exactly one sink metabolite (Bertsimas and Tsitsiklis, 1997), biomass can always be produced without concomitant secretion of the product. This establishes that the presence of an anchor reaction(s) is a necessary condition for growth-product coupling. The sufficient condition for the coupling arises when one of the split products of the anchor reaction becomes essential (or the most economic precursor) for growth, while another split product can, at least in part, only be channeled out through the production pathway (Fig. 1a).

## 3 Results

We show that a growth-product coupling necessarily requires an anchor reaction essential for growth that splits a substrate molecule to form two or more products (or parts of them) (Fig. 1a). Thus, the algorithms that design gene deletion strategies to create growth-product coupling (Burgard et al., 2003, Patil et al., 2005, Klamt and Mahadevan, 2015), essentially design network reductions that render an anchor reaction essential for (optimal) growth and that prohibit the utilization of one of the anchor reaction products for biomass synthesis. Due to their direct relevance for biorefining, we here focus on anchor reactions acting on carbon compounds. From a biochemical perspective, these can be broadly classified in two categories: I) those involving a C-C bond cleavage, and II) those involving a cleavage of a bond between carbon and a heteroatom. The latter category includes anchors engaging redox or energy co-factors. An excellent example of a coupling through such an anchor is the *natural* coupling between the cell growth and ethanol formation in *S. cerevisiae* under anaerobic conditions. In this case, ethanol formation is the optimal route for regenerating NAD^+^ needed for growth (Verduyn et al., 1990). A heterologous production example is a yeast mutant where 2,3-butanediol synthesis provides an optimal NADH sink (Ng et al., 2012).

We next consider biotechnological suitability of different biochemical sub-classes of anchor reactions involving carbon compounds. Anchors involving co-factors are tempting candidates for creating coupling since co-factors are hub metabolites being often associated with both growth and production pathways. On the minus, the global utilization of co-factors in any metabolic network means that a substantial network reduction is required to enforce the coupling. This is undesired as the resulting strain is likely to be less vital. Furthermore, the metabolic enzymes may quickly evolve to utilize alternative co-factors (e.g. NADH instead of NADPH) and thus break the coupling (Ellington and Bull, 2005, Hult and Berglund, 2007). Substantial adaptive evolution can be expected to be required after the network reduction to reach attractive productivities (Otero et al., 2013). Thus, calling for stable coupling designs in which cells are unlikely to escape the coupling by changing redox co-factor specificity.

Other reactions cleaving a bond between carbon and a heteroatom could also act as coupling anchors under some specific media conditions. For example, if a single amino acid is an essential ammonium source, a coupling could be created with a reaction cleaving the C‐N bond. However, C‐C cleaving anchors would work also under minimal medium conditions that are commonly used in industrial processes. Among the anchor reactions involving C‐C bond cleavage, those producing C1 compounds, such as CO_2_, as one of the cleavage products are not biotechnologically relevant except for carbon-fixing hosts. The remaining C‐C bond cleaving anchor reactions are broadly applicable for robust growth-product coupling.

To identify all biotechnologically relevant anchor reactions, we searched all the known biochemical reactions for C-C cleavage. We extracted, from the KEGG database (rel. 78, Apr 1st, 2016) (Kanehisa and Goto, 2000, Kanehisa et al., 2016), all reactions that act on a C-C bond between fully defined compounds (that do not include a C1-compound). There were 223 such reactions belonging to four main EC classes: 29 oxidoreductases, 52 hydrolases, 27 transferases, 92 lyases, and 23 reactions with no EC classification (Fig. 1b, Supplementary table 1). We filtered out reactions lacking genetic evidence, i.e. the reactions lacking KEGG KO annotation or a microbial gene annotation in BiGG reaction database (King et al., 2016). To further refine our catalogue of anchor reactions, we used ΔG estimates (see Supplementary information) and accordingly filtered out reactions that cannot, under physiological conditions, proceed in the direction of C-C cleavage and reactions for which thermodynamics were uncalculable (Fig. 1b, Supplementary table 1).

The remaining 97 anchor reactions were further filtered to remove methyl group exchange reactions, thiamine cofactor involving, and reactions carrying out cleavage of toxins and microbial cell biomass components such as sphingolipids (see Supplementary information for details). The anchors that cleave methyl groups or involve a thiamine cofactor are practically uninteresting because of their usage across several metabolic pathways. Further, toxin or biomass component degradation reactions are biotechnologically irrelevant in typical production conditions wherein the substrates are not available in quantities relevant to the total product titer. The remaining 62 anchor reactions mark attractive targets for biotechnology. These include some hydrolases and transferases, but mostly lyases (Fig. 1b, Supplementary table 1). Illustrative anchors include isocitrate lyase (Fig. 1c), and 3-fumarylpyruvate fumarylhydrolase (Fig. 1d). Indeed, one of the successful examples of growth-product coupling by now, succinate production in *S. cerevisiae*, is anchored by isocitrate lyase (Fig. 1e). In this strain, gene deletions targeting synthesis of L-serine from the usual route (starting in glycolysis) renders isocitrate lyase (anchor reaction) essential for growth (Otero et al., 2013). Applying the here introduced concept of anchor reactions, reduces the general problem of designing a growth-product coupling to identifying network modifications that make the appropriate anchor reaction(s) growth essential, and channel one of the split compounds to the desired product.

The 62 biotechnologically relevant anchor reactions are widespread among genome-sequenced species, with many harboring over 20 anchors (Fig. 1f, Supplementary information). We extracted the prevalence information by annotating protein sequences in KEGG database (rel. 78, Apr 1st, 2016) with eggNOG-mapper (Huerta-Cepas et al., 2016). For each prokaryotic and fungal protein in KEGG database, fine grained orthologs (i.e. discarding in-paralogs) across 2031 genomes were predicted. KEGG annotations from orthologous sequences were then propagated to each query, thus allowing us to re-annotate proteins without functional information in the original KEGG database (i.e. from non-model organisms). In total, we re-annotated 8,102,418 KEGG proteins from 2014 prokaryotic and 110 fungal species. After the re-annotation process, 47,464 KEGG proteins received KO assignments associated to C-C cleaving reactions, 8892 more entries than in the original KEGG database for the same set of organisms (38,572 proteins). EggNOG-mapper was executed in DIAMOND mode with an E-value threshold of 10^−3^. The information on the prevalence and the nature of anchor reactions in a species can be used in choosing an appropriate host for a given product, or as a genetic source for a suitable anchor reaction. Most anchor reactions are centrally located in the carbohydrate, amino acid, and energy metabolic pathways (Fig. 1g). Thus, they are likely to carry high fluxes, increasing their attractiveness for biotechnological exploitation. Furthermore, the metabolic centrality of the anchor reactions can be exploited to construct pre-reduced chassis strains where these are made essential. Such chassis strains can be used to plug-in different engineered pathways for product synthesis starting from the cleavage product(s). Furthermore, new heterologous production pathways could preferentially be designed starting with the products of the anchor reactions. Some species, including industrially important *E. coli,* harbor almost 40 anchor reactions making them attractive for chassis designs.

## 4 Conclusions

Overall, the identified C‐C cleaving anchor reactions constitute fundamental and universal targets for robust growth-product coupling and chassis construction. The coupling anchored in these reactions will also provide means to positively select for producer cells and hence holds potential for accelerating the development of new cell factories.

## Author contributions

Conceived the idea: PJ. Planned the research: PJ & KRP. Performed the research: PJ. Planned and performed the annotation of anchor reactions: JHC & PB. Analyzed the results and wrote the manuscript: PJ & KRP.

## Competing financial interests

The authors declare no competing financial interests.

## Appendix A Supplementary material

Supplementary material Supplementary table 1**.** Provided separately as Microsoft Excel file. Table caption: C-C bond forming and cleaving reactions, anchor reactions, (having reactants with defined atomic formulas and more than a single carbon in both cleavage products) extracted from KEGG database (rel. 78, Apr 1st, 2016) (Kanehisa and Goto, 2000, Kanehisa et al., 2016), with binary variables indicating whether the anchor reaction belongs to the ΔG filtered set due to having a reaction ΔG estimate (pH 7.0, ionic strength 0.1 M, equimolar reactant concentrations, from Equilibrator (http://equilibrator.weizmann.ac.il/; the 2nd of June 2016; taken from the downloadable file, or otherwise directly queried on the website (Flamholz et al., 2012)) lower than a liberal threshold of +30 kJ/mol in the direction of C-C cleavage, and another binary variable indicating whether the anchor reaction belongs to the final set of biotechnologically relevant anchor reactions after filtering out of toxin, microbial cell biomass component, and bound thiamine diphosphate cleaving reactions (pathway filtered).
