# Supplementary material for: Metabolic anchor reactions for robust biorefining
Source: Metab Eng. 2017 Mar;40:1–4. doi: 10.1016/j.ymben.2017.02.010 (PMC5368410; doi:10.1016/j.ymben.2017.02.010)
Supplement: Supplementary file 2 — Supplementary material [file mmc2.pdf]

Supplementary methods and results

Biotechnologically relevant anchor reactions were identified among all known biochemical reactions (i.e. a total of 10093 reactions) in KEGG database (rel. 78, Apr 1st 2016) (Kanehisa & Goto, 2000, Kanehisa *et al.*, 2016). All reactions acting on carbon-carbon (C-C) bonds that generate cleavage products having more than a single carbon and have fully defined atomic compositions for all reactants, were extracted from KEGG Reaction database, using information also from KEGG Compound and RPAIR databases. Reactions both forming and cleaving a C-C bond were considered because the reaction directionalities are mostly unknown and are concentration dependent. The extracted set of C-C cleavage reactions contained a total of 223 reactions with the following EC classifications: 29 EC 1.x.x.x, 52 EC 2.x.x.x, 27 EC 3.x.x.x, 92 EC 4.x.x.x, and 23 EC x.x.x.x (Supplementary table 1). Next, ΔG estimates of the reactions (under pH 7.0, ionic strength 0.1 M, equimolar reactant concentrations) in the extracted set were got from Equilibrator (http://equilibrator.weizmann.ac.il/; the 2nd of June, 2016; taken from the downloadable file, or otherwise directly queried on the website (Flamholz *et al.*, 2012)). Reactions with the ΔG estimate higher than a liberal threshold of + 30 kJ/mol (chosen based on fructose bisphosphate aldolase, reaction known to switch direction between glycolytic and gluconeogenetic state) in the direction of C-C cleavage were filtered out from the set of reactions. Reactions for which no ΔG estimate was possible to estimate were also filtered out. The resulting set of reactions considered thermodynamically feasible contained a total of 97 reactions with the following EC classifications: 2 EC 1.x.x.x, 25 EC 2.x.x.x, 17 EC 3.x.x.x, 52 EC 4.x.x.x, and 1 EC x.x.x.x (Supplementary table 1). Further, methyl group exchange reactions, thiamine cofactor involving reactions, and reactions carrying out cleavage of toxins or microbial cell biomass components such as sphingolipids, were filtered out. The resulting pathway filtered subset of the reactions contained a total of 62 reactions with the following EC classifications: 12 EC 2.x.x.x, 8 EC 3.x.x.x, and 43 EC 4.x.x.x (Supplementary table 1). The pie chart was generated using R v. 3.2.0 (Team, 2008), with the function 'ggplot' (Wickham, 2009).

Histogram of the prevalence of the final set of biotechnologically relevant C-C cleaving anchors in KEGG organisms (Figure 1g) was generated of the data found in the Supplementary table using R v. 3.2.0 (Team, 2008), with the function 'ggplot' (Wickham, 2009). Extraction of the data is explained in detail in the manuscript main text.

Finally, the localizations of the reactions (the final set of 62 reactions) in global metabolic network were identified and visualized using iPath v2 (Letunic *et al.*, 2008, Yamada *et al.*, 2011).

References

Flamholz A, Noor E, Bar-Even A & Milo R (2012) eQuilibrator--the biochemical thermodynamics calculator. *Nucleic Acids Res* **40**: D770-775.

Kanehisa M & Goto S (2000) KEGG: kyoto encyclopedia of genes and genomes. *Nucleic Acids Res* **28**: 27-30.

Kanehisa M, Sato Y, Kawashima M, Furumichi M & Tanabe M (2016) KEGG as a reference resource for gene and protein annotation. *Nucleic Acids Res* **44**: D457-462.

Letunic I, Yamada T, Kanehisa M & Bork P (2008) iPath: interactive exploration of biochemical pathways and networks. *Trends Biochem Sci* **33**: 101-103.

R Core Team (2016) R: A language and environment for statistical computing. R Foundation for Statistical Computing, Vienna, Austria. URL http://www.R-project.org/.

Wickham H (2009) *ggplot2: Elegant graphics for data analysis*. Springer-Verlag, New-York.

Yamada T, Letunic I, Okuda S, Kanehisa M & Bork P (2011) iPath2.0: interactive pathway explorer. *Nucleic Acids Res* **39**: W412-415.
